# Peer-learning and support among health policy and systems research actors in West Africa: a social network analysis

**DOI:** 10.1186/s12961-025-01417-6

**Published:** 2025-11-13

**Authors:** Selina Defor, Fidele Kanyimbu Mukinda, Fadima Yaya Bocoum, Ermel Johnson, Irene A. Agyepong, Uta Lehmann

**Affiliations:** 1 School of Public Health, University of the Western Cape, Cape Town, South Africa; 2 West African Network of Emerging Leaders in Health Policy and Systems (WANEL), Accra, Ghana; 3 Public Health Faculty, Ghana College of Physicians and Surgeons, Accra, Ghana; 4 Health Services to Systems Research Unit, South African Medical Research Council, Cape Town, South Africa

**Keywords:** Health policy and systems research, Social network analysis, Capacity-building, West Africa, Peer learning, Regional networks, Relational infrastructure

## Abstract

**Background:**

Health policy and systems research (HPSR) is vital for strengthening health systems, yet its development in West Africa remains constrained by limited capacity. To strengthen capacity, the West African Network of Emerging Leaders in Health Policy and Systems (WANEL) was created to foster peer learning and cross-country collaboration among early- and mid-career HPSR actors. This study used Social Network Analysis (SNA) to examine WANEL’s structure and functioning, with the aim of understanding how well the network supports its capacity and HPSR field-building goals.

**Methods:**

A cross-sectional whole-network survey was conducted with all 103 WANEL members, supported by document reviews and qualitative interviews. Relationship types assessed included acquaintance, communication, advice, mentorship and research collaboration. Data were analysed using Gephi to visualize relational patterns and compute metrics such as density and centralization, while qualitative findings provided context for interpreting network dynamics.

**Results:**

While WANEL has enhanced cross-country awareness and disciplinary diversity, the network exhibits low cohesion and high centralization. Key support relationships, particularly mentorship, advice and collaboration are sparse and unevenly distributed. A few actors dominate the flow of information and access to opportunities, while many, especially early-career and francophone actors, remain peripheral or isolated. Network interactions are driven by prior relationships and linguistic or professional affinity, limiting broader engagement.

**Conclusion:**

Findings reveal structural barriers that constrain WANEL’s potential to act as an inclusive platform for HPSR capacity-strengthening. To fulfil its vision, the network must address its current fragmentation by building stronger cross-cutting ties, broadening participation and decentralizing influence. This study contributes empirical insights into the design and governance of regional HPSR networks in low- and middle-income contexts and underscores the importance of relational infrastructure in advancing collective capacity.

**Supplementary Information:**

The online version contains supplementary material available at 10.1186/s12961-025-01417-6.

## Background

Health policy and systems research (HPSR) plays a critical role in developing health systems that effectively meet the health needs of their populations [1]. The context-specific nature of health systems reinforces the importance of local knowledge and highlights the need for context-embedded HPSR to support health system development. However, the number of individuals, groups, or organizations actively conducting HPSR, especially in low- and middle-income countries (LMICs) remains limited [2–4]. As in much of sub-Saharan Africa, the HPSR landscape in West Africa has long been characterized by limited capacity to generate research evidence for decision-making and health system strengthening [5, 6]. This capacity gap, though varying across countries [7], is particularly critical in countries where HPSR remains in its embryonic stage [8].

Although the HPSR literature emphasizes the need for capacity-strengthening [9–11], much of it focuses on selected elements such as evidence-informed policy-making [12] or organizational strategies [4]. Recent efforts have proposed core competencies for HPSR training [13], while several others have documented empirical experiences of capacity assessments and strengthening efforts [14–19]. However, comprehensive frameworks to guide a holistic and synergistic approach to HPSR capacity development remain scarce [20]. To address this gap, Mirzoev et al. [20] recently proposed a comprehensive system-oriented framework for strengthening HPSR capacity. The framework reflects the applied, cross-disciplinary and multi-actor nature of HPSR, emphasizing the importance of synergies among researchers, practitioners and advocates. It highlights the need to bridge the worlds of research, practice and advocacy, with particular emphasis on strengthening collective linkages among individuals and organizations [20]. This aligns with other HPSR scholarship advocating for the creation of integrated HPSR communities and networks within and across countries to advance the field and promote the use of research evidence in policy-making processes [3, 5, 9]. Networking facilitates knowledge exchange, peer support, mentorship and collaborative research initiatives [21–23] and can enable individuals and institutions to collectively build and share expertise [24]. Despite calls for greater investment in such networks to support HPSR capacity in LMICs [3, 25], empirical insights into how these networks function, how relationships are formed and sustained and how they contribute to capacity development remain limited. In the field of public health, Mays and Scutchfield [26] sought to fill this gap using a systems science lens. They used three key structural characteristics – breadth, density and centrality – to assess network structure and functionality on the basis of diversity, connectivity and influence [26]. Retrum et al. [27] expanded on this work to further explore the impact of network structure on collaborative engagements in the field of public health [27]. This paper draws on these insights to analyse the structural properties of the West African Network of Emerging Leaders in Health Policy and Systems Research (WANEL), with the aim of understanding how and why the network functions as a strategy for building HPSR capacity. WANEL was established to strengthen HPSR in West Africa by fostering peer learning and peer support, cross-disciplinary exchange and cross-country collaboration. Members represent a range of HPSR-related disciplines and backgrounds from 11 of the 15 countries in the sub-region. By offering a platform for interaction and mutual support, WANEL aspires to strengthen both individual competencies and collective HPSR capacity for the West Africa. However, as Mirzoev et al. [20] posit, this individual and collective capacity goal realization is conditioned by the strength and quality of relationships among the diverse actors. Relationships serve as connective tissue that enables capacity at one level to support or influence another. Thus, understanding the relational dynamics within WANEL – how members connect, collaborate and support one another – is therefore crucial to enhancing the network’s effectiveness and sustainability.

This present paper uses Social Network Analysis (SNA) to explore the connectivity structure and functioning of WANEL. SNA is a set of theories, tools and techniques that facilitate a better understanding of the structure of a social network and the interactions within it [28]. SNA has been extensively used in a variety of ways to assess networks in diverse fields: It has been employed in the analysis of interaction patterns to identify the strengths and weaknesses of network operations to support a planning process to improve interactions [29, 30]. As a reflective tool, network maps or sociograms from SNA have been used to raise awareness about the nature of a network for a collective assessment of the degree of sustainability of the network [31]. SNA has also been used as a planning tool to identify gaps within networks and to strengthen connections to better integrate isolated members and strengthen the capacity of the network to act collectively [32]. In addition to analysing patterns of relationships in a network, SNA has also focused on the examination of the availability of resources and the exchange of these resources between network actors. For research collaboration assessments, SNA has been employed to highlight barriers and enablers to interdisciplinary research within and across institutions [33–35]. We applied SNA in this paper to examine the WANEL’s connectivity structure and its implications for the network’s functioning and sustainability. This paper is part of a broader study that aims to understand and inform the development and functioning of sub-regional HPSR networks. In two previous papers, we described the history of WANEL [7], and its early impact, detailing the activities that facilitated connections among some emerging HPSR actors and their self-reported impact of the activities on their career development in the field.^1^ A forthcoming paper provides further details of the contextual factors, design and implementation gaps that impacted on broad member connectivity and the network’s overall effective functioning.

**Fig. 1:**
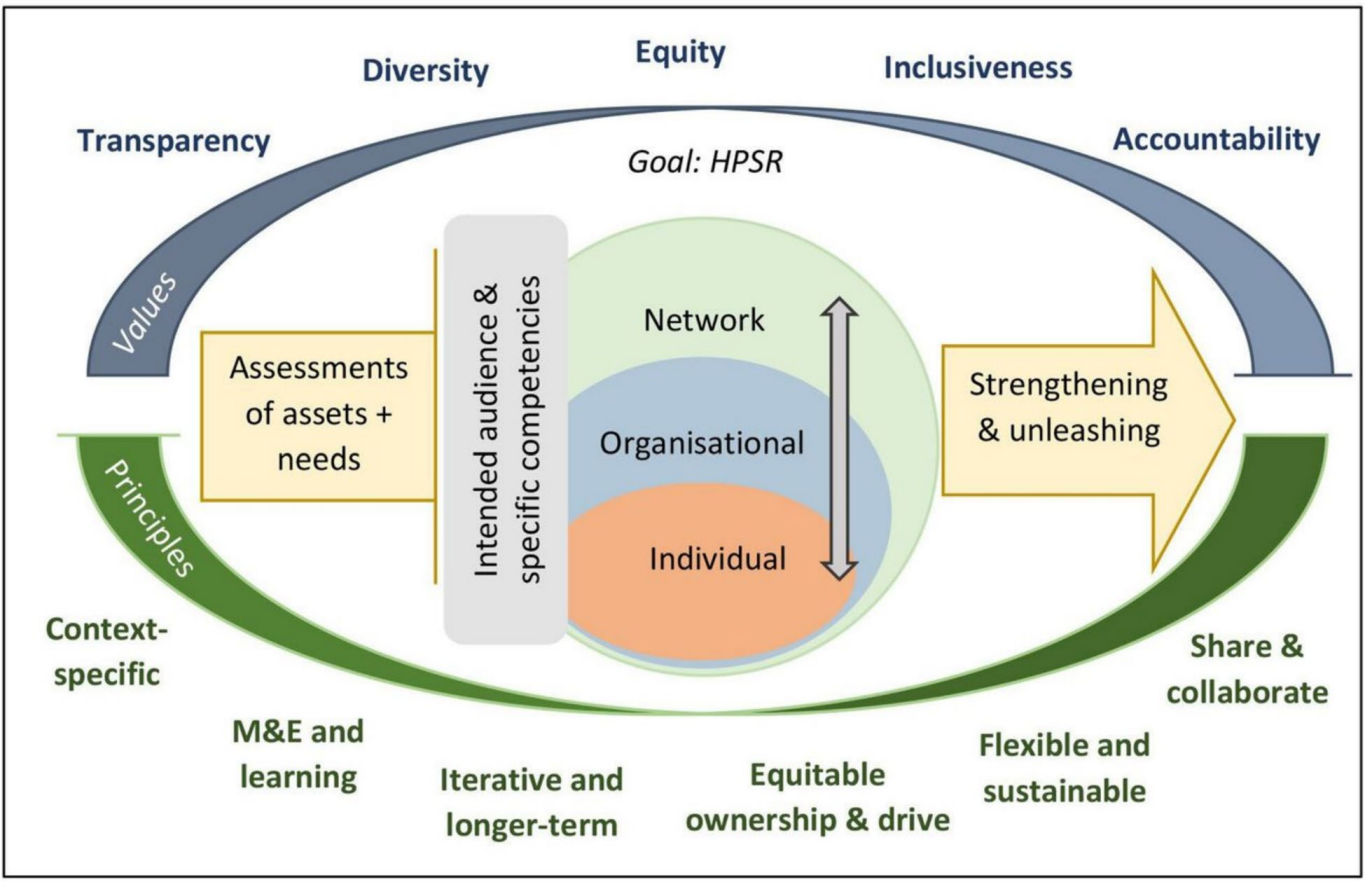
Conceptual framework for systemic capacity-strengthening for health policy and systems research Source: Mirzoev et al. (2022)

This present paper provides empirical insights into the relational fabric of WANEL and how this contributes to the network’s functioning and sustainability. It draws on Mirzoev et al.’s conceptual framework for systemic capacity-strengthening (Fig. 1) to analyse the network’s structure to understand how patterns of connection and interaction shape its ability to function as a platform for HPSR development. The framework identifies three interrelated levels of capacity (individual, organizational and network levels) and emphasizes the amplification potential and blurred boundaries across these levels. It advocates for intervention to begin with assessment of capacity assets and needs to tailor the efforts to diverse actors while leveraging existing resources. It also highlights the importance of addressing power dynamics to establish values and principles to ensure inclusivity and equitable ownership.

## Methods

### Aims

This paper seeks to analyse the connectivity structure of WANEL. It maps the overall network structure and key relational patterns among network members to identify central actors, isolated members and sub-group dynamics; it assesses barriers to interaction and collaboration; and it provides recommendations to strengthen the network’s capacity to foster peer learning and collective action for systemic HPSR capacity-strengthening.

### Study design

This was a cross-sectional network study that drew on what network scholars describe as an “ethnographic sandwich” [36], in which qualitative methods are conducted before and after the SNA survey to inform the design, interpretation and validation of network findings. In line with this approach, the study was embedded within two qualitative processes. First, a document review and key informant interviews were conducted to define the boundaries of the network, identify the network membership characteristics and understand the theory of change underpinning WANEL’s formation and operations. These early steps also helped establish conceptual clarity about which relational mechanisms were most relevant to measure, thereby enhancing the construct validity of network ties. A series of post-SNA stakeholder engagements, including in-depth interviews with isolated members and a validation workshop, allowed for contextual interpretation of the findings and triangulation across data sources to strengthen validity. This paper focuses mainly on the structural properties of the network.

### Study population

We used a “whole network” design, which examines the relationships among all actors in a defined group [37]. The study population comprised all 103 registered members of WANEL as of January 2020. The network directory served as a roster from which respondents selected members with whom they had connections, ensuring that all potential ties were systematically represented. This roster-based approach was adopted to enhance measurement reliability by minimizing respondents' recall error and reducing concerns about informant accuracy [36].

### Tool design and data collection

An extensive document review began in 2019 and culminated in the articulation and validation of WANEL’s theory of change in 2020 through individual consultative meetings with 22 stakeholders. In SNA, the types of relationships measured are defined by the researcher, in consultation with network stakeholders, to ensure alignment with the project’s goals and the characteristics of the group under study [37]. The relational mechanisms identified as central to WANEL’s goals, including information sharing, peer support (including mentorship and coaching) and collaborative research, were co-defined with stakeholders and aligned with the network’s theory of change to enhance validity. These categories informed the design of the survey tool, which was first developed in English in 2021. The instrument was piloted with three bilingual members in February 2022 to assess clarity, consistency and relevance. On the basis of feedback, several items were revised, including the addition of explicit definitions of each tie in the survey preamble to reduce interpretation bias and strengthen reliability. The French version of the instruments was then piloted with two francophone members to ensure inclusivity and minimize measurement bias across the bilingual network. The final survey included closed-ended questions organized around network relationships and respondents’ characteristics. Relationship questions focused on communication/information sharing, advice-seeking, mentorship and research collaboration, mirroring the core components of the WANEL theory of change. Participants were also asked to indicate whether they knew other HPSR actors before joining WANEL. Respondents’ characteristics included country of residence, professional background, year of membership, discipline, academic qualifications, years of professional experience, age and gender.

### Data collection

Data collection involved a self-administered online survey in English and French. Participants identified WANEL members: (1) who they knew prior to joining, (2) who became acquainted with after joining, (3) with whom they shared HPSR-related information (and the frequency), (4) from whom they sought professional advice, (5) from whom they received mentorship and (6) with whom they engaged in joint research. For communication ties, participants reported interaction frequency on a standardized scale (biannually, per semester, quarterly, monthly, weekly, daily, or never). As Wasserman and Faust (1994) observed, sociometric questions using ratings or full ranking orders tend to be more reliable than fixed designs with limited response categories [36]. Thus, the standardization of communication options further strengthens reliability by ensuring comparability across respondents. For this “whole network” design, we considered a relationship present if a respondent reported it, which also allowed us to capture ties involving non-respondents. To ensure data quality, reported ties were systematically checked for errors and inconsistencies, and respondents were notified of errors and given sufficient time to rectify discrepancies prior to analysis. These measures were taken to ensure that the relational data accurately reflected the realities of the network.

### Data analysis

Data were exported from the online survey platform and cleaned in Microsoft Excel ^®^ 365 to address missing values, standardize member identifiers and prepare adjacency matrices for network analysis [37]. Separate matrices were created for each relationship type: acquaintance, communication, professional advice, mentorship and research collaboration. These matrices were saved as comma-delimited values (.csv) and imported into Gephi software version (0.9.7) for network visualization and computation of the network’s matrices. In-built algorithms in Gephi allowed for the visualization and the computation of network structural properties. It enabled the generation of network graphs and the calculation of centrality, density and centralization matrices of the network to determine (1) the level of cohesion and on-going peer-support in the network, (2) the degree of actor embeddedness in the network and (3) the network’s resilience and chances of sustainability. Sociograms (network graphs) were generated to visually represent network structures, with nodes colour-coded by language, discipline, educational level and professional background. Network actors were represented by a coded node, and relations between actors were denoted with an arrowed directed line (edges) for directed relationships. The size of the node depended on the number of connections (degree centrality) or the number of times an actor was sitting on the shortest path between two actors (betweenness). The visualization allowed us to identify not only influential central actors that are the most connected but also peripheral actors as well as actors with no connections (isolates). See Box 1 below for the definition of network properties explored. The graphs were generated to depict the different forms of relationship within the network, namely pre-WANEL and current acquaintance, information sharing/communication, peer-support (advising and mentorship) and collaborative research activities. Additional graphs were also generated for sub-networks such as the WANEL thematic working groups (TWGs).

Qualitative analysis complemented the quantitative findings. Key informant interviews and discussions from post-SNA stakeholder engagements were coded thematically to capture perceptions of network functioning, barriers to interaction and suggestions for strengthening ties. These qualitative insights were used to contextualize the SNA metrics and explain observed patterns, following the ethnographic sandwich approach [38]. The reporting guidelines for social networks in health research [39] was used in reporting this study. All responses were confidential, and participants were informed that their individual data would be anonymized during analysis. Ethical approval for the study was secured from the University of the Western Cape (Ref No. BM20/4/16) and the Ghana Health Service Ethics Review Committee (Ref No. GHS-ERC015/03/20). Informed consent was obtained from all respondents.

Box 1. Definitions of relevant network-level and node-level measures explored.
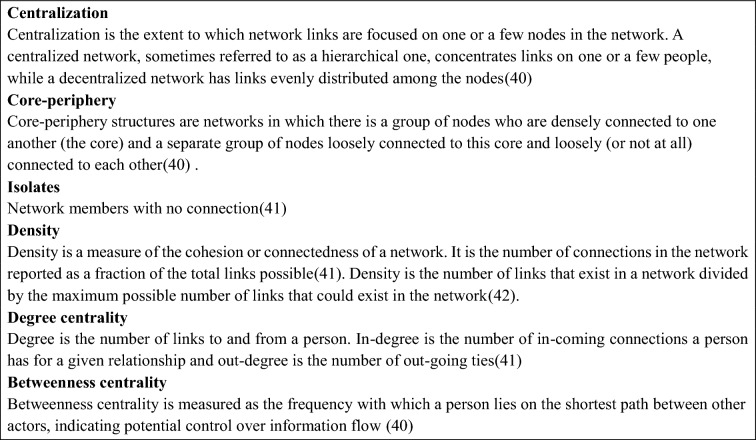


## Results

### Characteristics of respondents

Of 103 members, 94 completed the survey (91% response rate), comprising 66 anglophone and 28 francophone respondents. The network demonstrated strong disciplinary diversity: 29.79% from medical sciences, 26.60% from public health, 9.57% from social sciences and 8.51% from other related disciplines. Professionally, network members represented a mix of HPSR evidence production and its application, involving researchers (31.91%), medical practitioners (21.28%) and non-medical practitioners (14.8%), including policy advocates. Although WANEL is considered a network of emerging leaders, about 26% of its current membership comprises of senior actors with over 10 years of professional experience in their fields of endeavour. Furthermore, membership distribution is heavily skewed towards Nigeria (46.81%) and Ghana (22.34%), with the two countries alone constituting about two thirds of the network. Table 1 describes the characteristics of the network actors in more detail.

**Table 1:** Characteristics of respondents

Gender	*N* (%)
Male	51 (54.26)
Female	43 (45.74)
Age	
51–60 years	6 (6.38)
41–50 years	35 (37.23)
31–40 years	48 (51.06)
20–30 years	5 (5.32)
Country of origin	
Nigeria	44 (46.81)
Ghana	21 (22.34)
Benin	13 (13.83)
Burkina Faso	4 (4.26)
Niger	3 (3.19)
Togo	3 (3.19)
Senegal	2 (2.13)
Ivory Coast	2 (2.13)
The Gambia	1 (1.06)
Mali	1 (1.06)
Highest level of education	
PhD	37 (39.36)
Master’s	53 (56.38)
Bachelor’s	4 (4.26)
Disciplinary background	
Medical sciences/medicine	28 (29.79)
Global health/public health	25 (26.60)
Social science/sociology	9 (9.57)
Health economics/economics	8 (8.51)
Other related disciplines	8 (8.51)
Health policy and management	7 (7.45)
Medical anthropology	5 (5.32)
Communication science/media studies	4 (4.26)
Professional background	
Researcher	30 (31.91)
Medical practitioner	20 (21.28)
Doctoral researcher	14 (14.89)
Non-medical practitioner	14 (14.89)
Lecturer	12 (12.77)
Post-doctoral researcher	4 (4.26)
Years of professional experience	
Over 10 years	25 (26.60)
8–10 years	13 (13.83)
4–7 years	27 (28.72)
1–3 years	24 (25.53)
6 months to less than 1 year	2 (2.13)
Less than 6 months	3 (3.19)
Cohorts	
2019	26 (27.66)
2018	16 (17.02)
2017	18 (19.15)
2016	12 (12.77)
2015	22 (23.40)

## Network-level metrics

### Overall network structure

#### From acquaintance to actual interaction

**Fig. 2:**
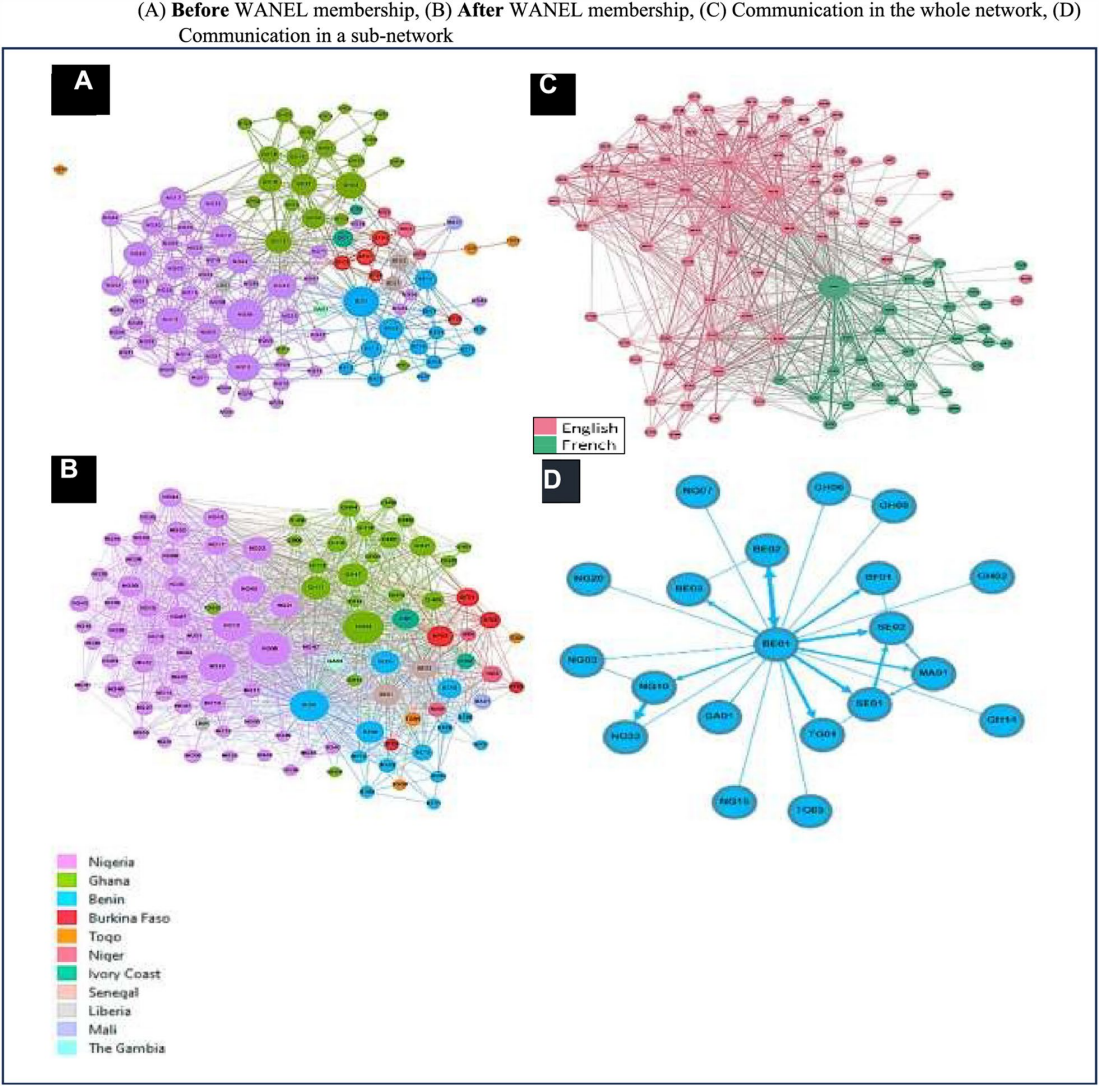
Knowledge and communication patterns in WANEL: **A** Before WANEL membership, **B** after WANEL membership, **C** communication in the whole network and **D** communication in a sub-network

We calculated two network-level indices – density and centralization – to measure the level of cohesion among members and determine the extent to which the cohesion is organized around specific actors or sets of actors. Across the six relationships examined in the network analysis, we observed a varying density of connections across the different relationship forms, moving from mere acquaintance to more intensive interactions and peer-engagements that support capacity-strengthening in networks: network density for pre-WANEL acquaintance connection 6.3% (*n* = 662), current acquaintance connection 19.5% (*n* = 2050), communication connection 10.6% (*n* = 1114), advice connection 4.0% (*n* = 424), collaboration connection 2.3% (*n* = 242) and mentorship connection 1.0% (*n* = 106). A possible connection of 10,506 is expected for a network of 103 nodes with directed ties, if every member was connected to each other for each of the relationships under examination. With an actual connection of 2050, the highest density is reported for current acquaintance at 19.5%, followed by communication at 10.6%. Transitioning from a density of 6.3% to 19.5%, the acquaintance networks (Fig. 2A, b) depicting WANEL members’ awareness of other West African emerging HPSR before and after joining the network demonstrate an increased level of awareness with the formation of WANEL. However, this density drops as we move from mere acquaintance to concrete interaction. The communication network (Fig. 2C), depicting the frequency of information and HPSR resource sharing among the emerging actors, has a density of 10.6%, which clearly shows that the increased awareness of the existence of other HPSR actors is not necessarily translating into actual engagement. Furthermore, a graph (Fig. 2C) also shows that communication is happening among members from similar linguistic backgrounds, with actor BE01 bridging the two linguistic blocks.

#### Cross-country and in-country connections before and after WANEL membership

The network graphs A and B in Fig. 2 compare WANEL members’ awareness of and acquaintance with other emerging HPSR actors within their respective countries and across West Africa before (Fig. 2A) and after involvement in the network (Fig. 2B). The network actors are clustered, and colour coded by country of origin. Graphically, graph B (current acquaintance) shows a more densely populated network structure than graph A (pre-WANEL acquaintance). The country connectivity chart [Fig. S1 in Electronic Supplementary Material (ESM) 1] further highlights the fact that the formation of WANEL has significantly improved cross-country connections (acquaintance) among emerging HPSR across West Africa. Members from francophone West African countries particularly reported increased cross-country connections with other actors from both anglophone and francophone countries. From zero connection, WANEL members in Mali and Togo reported 15.69% and 8.33% cross-country connections, respectively (Fig. S1 in ESM 1). These previously completely siloed HPSR actors now report current connections with HPSR actors in more than seven countries, including Ghana and Nigeria. Members from Cote d’Ivoire who were connected to HPSR actors in only three countries also reported current connections in ten WANEL member countries, including the Gambia, Ghana, Nigeria and Liberia. Similarly, with 37.13% connections, reports from Senegal also indicate expansion of pre-WANEL connections from only two countries to seven more countries, making Senegal the country with the highest level of cross-country connections. Fewer cross-country connections were reported for the anglophone West Africa countries except for the Gambia, which reported a 3.92% connectivity, extending their pre-WANEL connection from only Benin to now include connections in Ghana and Nigeria.

Unlike the cross-country connectivity pattern, the in-country HPSR actor connectivity reflects a rather mixed pattern. Benin, Burkina Faso, Ghana and Niger slightly deepened their in-country connectivity with the formation of WANEL, but Senegal and Togo recorded no further connection beyond their 55.56% and 100% pre-WANEL intra-country connection. In Togo, the third actor, node TG03, remained isolated from colleagues in-country, nodes TG01 and TGO2. The impact of the hugely disproportionate membership distribution observed earlier is highlighted in Mali and the Gambia, who reported zero in-country connection pre-WANEL and after WANEL formation, as the countries have been represented by a single actor each since the inception of the network. With a more than 50% increase in connectivity, Nigeria reported the strongest in-country connection with the formation of WANEL, at 31.84%.

#### Cross-professional interaction within WANEL

Another important finding is the budding cross-professional relationships within the West African HPSR community, even though regular communication appears to be happening primarily among members of the same professional backgrounds. HPSR lecturers reported the highest communication frequency at 19.70%, followed by the researchers 15.50% and medical practitioners 15.40%. The network seems to have enabled emerging communication and engagement across professionals (Table 2) and disciplinary backgrounds (Fig. S1 in ESM 2) to start nurturing a relationship to bridge the gap between HPSR evidence producers and evidence users, a pre-condition to WANEL’s vision for knowledge and evidence co-production for action.

**Table 2:** Density table of relationships: cross-professional communication patterns in WANEL

To From	Researcher	Medical practitioner	Non-medical practitioner	Doctoral researcher	Lecturer	Post-doctoral researcher
Researcher	15.50%	2.90%	2.90%	3.00%	3.70%	1.00%
Medical practitioner	2.30%	15.40%	2.60%	2.50%	1.30%	0.10%
Non-medical practitioner	1.30%	1.70%	7.80%	2.00%	0.80%	0.80%
Doctoral researcher	3.10%	3.10%	3.40%	10.50%	3.10%	1.10%
Lecturer	3.70%	2.40%	2.10%	3.00%	19.70%	1.50%
Post-doctoral researcher	5.00%	0.00%	0.60%	0.50%	1.10%	0.00%

## Node-level metrics

### Influential actors in WANEL

We calculated network members’ degree centrality (members’ in-coming and out-going connections) to identify actors with the highest connections and betweenness centrality (Box 1) to identify the most strategically positioned actors within WANEL. As shown in Fig. 2C above, communication within WANEL is mainly among members from similar linguistic backgrounds, with actor BE01 connecting the two linguistic blocks. This actor has an extremely high betweenness centrality score (Fig. S2, ESM 1), and appears to be controlling the flow of information in the network. Interestingly, not only does this node provide the bridge that connects the francophone and anglophone communities of emerging HPSR actors, but he also plays a central role in the network’s singular thematic working group (TWG), set up to support the resource and information needs of francophone actors (Fig. 2D). His positional advantage (control over information flow) is unparalleled by the main coordinator of the TWG (node NG10) who ironically appears to be occupying a rather peripheral position in this network (Fig. 2D).

### Peer support and collaboration patterns within WANEL

**Fig. 3:**
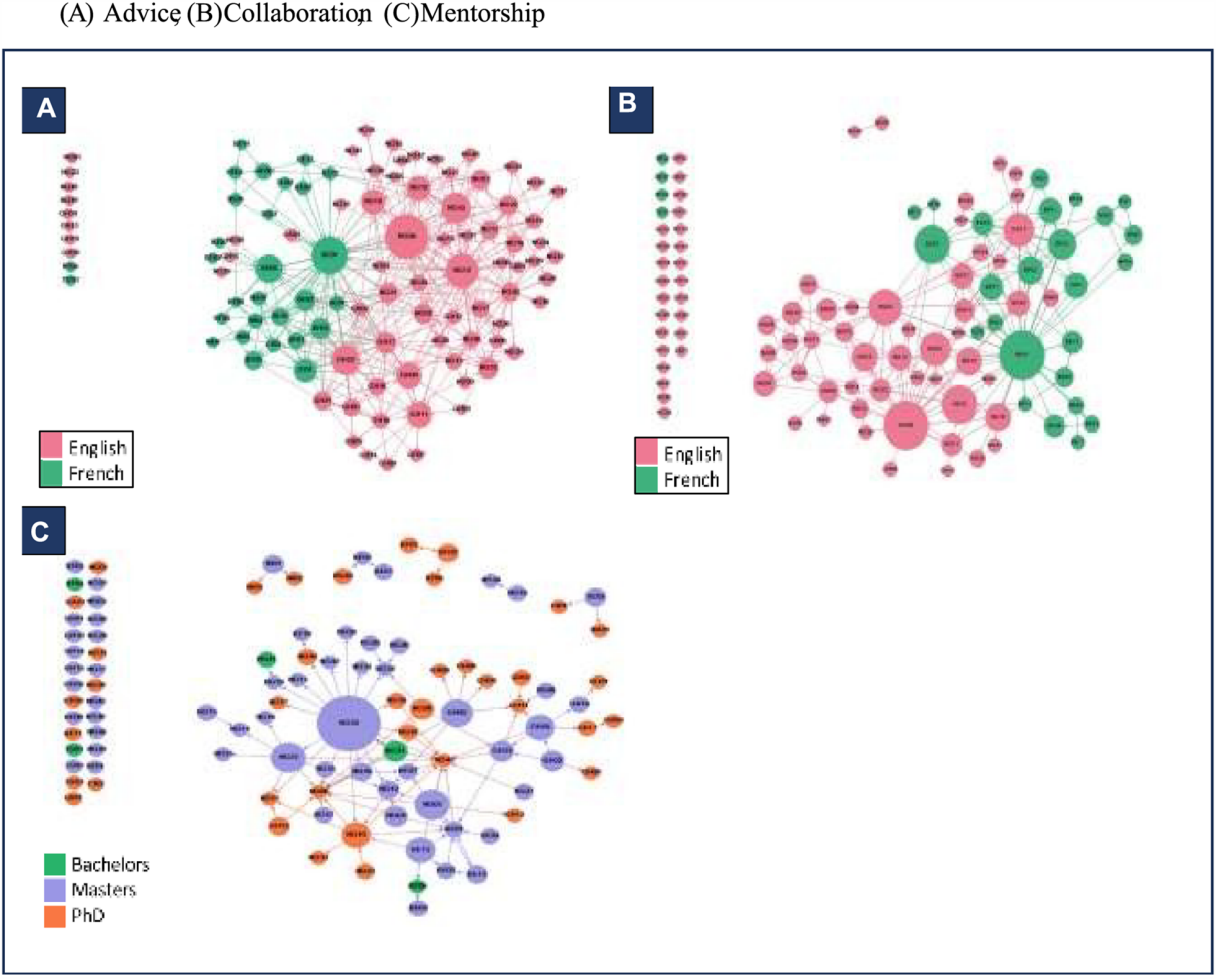
Peer support and collaborative activities among WANEL members

Figure 3 presents the graphs of the advice (A), collaboration (B) and mentorship (C) networks. The actors in the advice and collaboration network are clustered and colour-coded by linguistic backgrounds, while the mentorship is colour coded by the actor’s highest level of education. In all three networks, the large nodes (having the highest in-degree centrality scores) represent the most influential actors who are highly connected to other actors as go-to actors for HPSR-related advice and peer mentorship and as collaborating actors for diverse joint research activities. All three networks are characterized by a few densely connected nodes at the core of the network, with more sparsely connected peripheral actors and some totally isolated actors. All three networks recorded density scores that fall below 5%, showing a further drop in the communication density score. WANEL key players cut across these three networks. The first ten [10] actors with the highest in-degree scores in the advice network for instance include nodes NG08 (29), BEO1 (23), NG12 (20), NG43 (17), GH22 (16), BE05 (15), GH04 (13), GH11 (13), NG19 (12) and NG10 (12). Apart from node GH04, the same set of actors appear as central players in the mentorship network. Of these, only nodes BE01 and BE05 are from francophone West Africa (Benin), with the remaining eight (8) originating from Ghana (3) and Nigeria (5), respectively, which again highlights the under-representation of the francophone actors in WANEL.

### Pre-WANEL relationships drive advice and mentorship connections

**Fig. 4:**
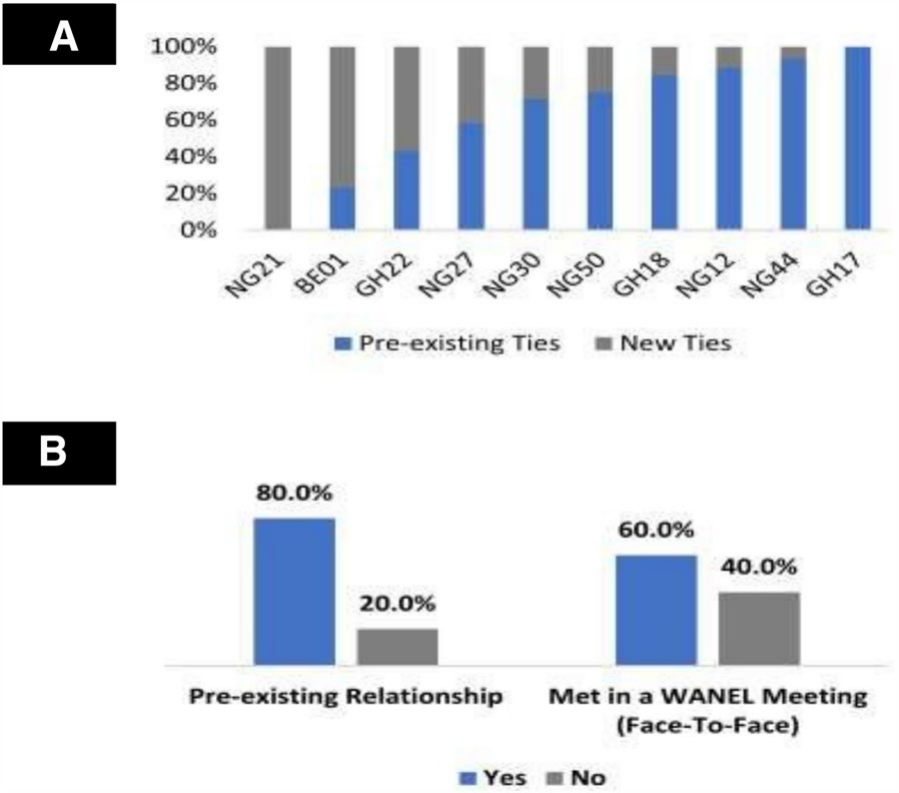
Peer coaching and peer mentorship patterns in WANEL. **A** Pre-existing and new ties for the top ten WANEL members with the highest out-degree scores in the advice -seeking network. **B** Relationship formation pattern of the cliques in the mentorship network.

Very importantly, it appears that the most active advice-seeking and mentorship relationships seem to revolve around relationships that existed before WANEL. Figure 4A below shows that the first ten members with the highest out-degree (advice seeking) centrality scores interact mostly with WANEL members with whom they shared a relationship before their WANEL membership. Similarly, in the mentorship network (Fig. 3C), an interesting pattern of intra-network fragmentation (clique formation) is observed. Further analysis shows that about 80% of the mentee–mentor connections among these cliques are based on pre-existing relationships (Fig. 4B).

### Isolated actors

Finally, the low levels of interaction are further heightened by the number of members who are totally isolated from on-going meaningful capacity-enhancing interactions in the three networks (advice 10, collaboration 28, mentorship 29). About 40% of the isolates are early-career HPSR actors (1–3 years’ experience) and another 40% are mid-career (4–7 years’ experience) (Table S1 in ESM 3). These isolated individuals could both benefit from and contribute to knowledge exchange, but they remain excluded from the advice network. A similar pattern is observed in the mentorship network, where about 40% of the PhD holders and about 26% of the members with the highest professional working experiences appear inaccessible to the early-career actors (Table S1 in ESM 3).

### Network centralization

Beyond the network’s asymmetric distribution by country, the network’s centralization score also stands at 66.08%, which appears quite consistent with the observed unequal levels of actor embeddedness depicted by the concentration of network connections among a few active and popular actors, as well as the limited relational paths and linkages among actors indicated by the extremely high betweenness centrality score of actor BE01.

## Discussion

The West African network of emerging leaders in health policy and systems (WANEL) aims to strengthen HPSR in the region by enabling peer learning, cross-disciplinary exchange and collaborative engagement across countries. The topology of a network shapes how information flows, how resources are distributed and how effectively a network achieves its goals, given that different structural patterns produce different outcomes, each with unique advantages depending on the network’s objectives. Through the lens of Mirzoev et al.’s framework for systemic capacity-strengthening in HPSR, we (a) illustrate WANEL’s rich potential to bridge individual, organizational and network capacity; (b) identify structural gaps that constrain this potential; and (c) make recommendations to enhance WANEL’s operation as a platform for system-wide capacity-strengthening.

### WANEL member composition: A rich constellation of HPSR actors

The embryonic network activated in 2015 with 29 emerging leaders comprising 12 PhD students, 10 master’s degree holders and 7 post-doctoral researchers [7], has grown into a rich West African HPSR learning community with about 40 HPSR actors with PhDs and 53 master’s degree holders, many of whom are currently pursuing doctoral training, representing varied disciplinary perspectives necessary for development of the field of HPSR [12, 43]. The diversity ranging from master’s students to postdoctoral researchers and early-career faculty, endows the network with the potential for peer-to-peer learning but also cross-generational learning and mentorship. Beyond the potential for individual capacity-strengthening through knowledge sharing and skills transfer, WANEL’s layered member composition also reflects what Mirzoev et al.’s describes as overlapping boundaries and amplification potential that cuts across the three levels of capacity-strengthening [20]. In addition to the disciplinary and experiential diversity, the network’s inclusion of both researchers and research evidence users presents a great potential for synergistic interaction that supports knowledge translation [44–48]. The interdisciplinary and cross-sectoral composition creates a good foundation for bidirectional knowledge exchange, allowing research to be informed by real-world needs while facilitating the translation of evidence into policy and practice. This creates an ecosystem that fosters collective learning and co-creation of context-specific solutions. Although West African countries differ in many respects, they share common health systems challenges [49]. WANEL’s design as a cross-country, cross-disciplinary and cross-sector network positions it well to facilitate the diffusion of contextually relevant HPSR knowledge and practices to enhance individual expertise and institutional capacity across borders. Studies have shown that interpersonal relationships often evolve into inter-organizational collaborations, generating ripple effects that strengthen environmental capacity [50]. Our earlier research on WANEL’s initial cohorts demonstrated how individual connections later translated into institutional collaborations involving Ghana, Benin and Togo.^2^ This progression exemplifies Mirzoev et al.’s concept of systemic capacity-strengthening and reinforces the importance of strategically leveraging synergies across levels. As interpersonal connections deepen, they not only enhance individual capacity but also lay the groundwork for sustained cross-country collaboration that will contribute to long-term field-building for HPSR in West Africa. However, WANEL’s ability to fully realize this potential is constrained by the network’s current connectivity structure. Mirzoev et al. argue that, given the applied, cross-disciplinary and multi-actor nature of HPSR, capacity-strengthening efforts must ultimately foster close engagement and synergistic collaboration across the diverse actor groups. In the next section, we examine WANEL’s member cohesion and embeddedness, and their implications for supporting systemic capacity-strengthening across levels.

### Network cohesion: Constraining knowledge sharing, peer support and collective action

As revealed by the social network analysis, WANEL exhibits low overall cohesion characterized by low density, high centralization and sparse interaction patterns, particularly in the advice and mentorship networks (Fig. 3a, c). Low-density networks limit members’ access to resources and opportunities for peer learning through knowledge sharing and information flow [40, 51]. Research shows that strong network cohesion enables sharing of more relevant tacit knowledge including people’s experiences, know-how and other complementary expertise that cannot be codified or captured in documents [27, 34, 52–54]. While WANEL’s communication network showed a density of 10.6%, Parise (2007) observed that networks of a similar size as WANEL typically demonstrate effective knowledge with densities between 15% and 20%. Similarly, strong interpersonal connections, built on a history of interaction [55], frequent communication and emotional attachment [56], have been shown to fosters social diffusion [40], knowledge transfer and peer-support [52, 57, 58], even among actors with dissimilar background [52]. This includes fostering norms of reciprocity, frequent interaction [52] and trust-building that support effective knowledge translation (KT) [59]. According to Lavis et al. [60], KT is most effective when sustained “linkage and exchange” exists between knowledge producers and users, a condition not merely met by co-presence in a network but by the quality and frequency of their interactions. A recent survey of members of the Health Systems Global community echoes this, identifying the lack of opportunities for meaningful member interaction and exchange as a key factor limiting the effectiveness of networks as capacity-building mechanism [61]. Within WANEL, communication tends to occur among actors from similar professional backgrounds (Table 2), limiting cross-sector engagement and, therefore, opportunities for knowledge co-production and translation. Evidence suggests that, as networks grow in size and complexity, maintaining high levels of connectivity becomes increasingly difficult [27, 40]. For example, a study of the Emerging Leaders for Global Health (EV4GH) network found that, although the network begun promoting intra- and cross-cohort mentorship after the early “ventures”, this became difficult to sustain as the network grew. Moreover, francophone members felt that peer-to-peer mentorship was more accessible to the anglophone members [62]. Thus, the member diversity in WANEL represents both a strength and a challenge; while broadening perspectives, it also creates structural barriers to interaction that limit the network’s overall cohesion and functionality. Realizing its capacity-building goal will require putting in place deliberate structures to facilitate peer-to-peer mentorship. The importance of such intentional effort is reflected in Nxumalo’s insights on HPSR in Africa and her experience with the Emerging Leaders Programme (ELP) of the Consortium for Health Policy and Systems Analysis in Africa (CHEPSAA) initiative. She emphasized the need for a more systematic mentorship models and schemes to support the growth of emerging researchers and the development of future leaders in HPSR in Africa [63].

### Actor embeddedness: Implications for network effectiveness and sustainability

Embeddedness describes the extent to which an actor is integrated into the fabric of a network, influencing their access to resources, opportunities for collaboration and ability to exert influence [64, 65]. WANEL’s current structure shows a pattern of unequal actor embeddedness and participation as revealed by individual members’ degree and betweenness centrality measures. For instance, the acquaintance network has a seemingly high density of 19.5%; however, this density is skewed by the disproportionately high degree centrality scores of just a few actors. The central actors are repeatedly identified as the go-to individuals for advice, peer mentorship and collaborative research. The key actor distribution, as depicted by the first ten actors with the highest degree centrality scores and the isolation of other equally competent and qualified members, could be an indication of the absence of widespread internal awareness and recognition of member expertise and competences. A similar pattern was observed in the EV4GH network study, where collaboration among network members was limited because members were often unaware of others in the network who shared their interests or geographical locations [62]. As Borgatti and Cross (2003) argue, people in a network can exploit the knowledge of other members only to the extent to which they are aware of “who knows what” and “who knows who knows what” [66]. Interestingly, these go-to actors were found to be “old friends” of their support-seeking colleagues who already had a certain degree of awareness and trust in their HPSR skills and competences. Competence-based and vulnerability-based trust are described as pre-conditions to advice-seeking in networks in the literature [41, 67, 68]. Consequently, it was unsurprising to discover that about 90% of the first ten advice seekers with the highest out-degree centrality scores had a pre-existing relationship with their alters before their WANEL membership, which explains the level of awareness of and confidence in their skills and expertise.

From a sustainability perspective, unequal embeddedness presents several risks. The overreliance on a small group of central actors makes the network vulnerable to disruption, particularly if these individuals disengage or experience burn out [41]. The EV4GH study similarly highlighted this challenge, noting that, while over dependence on a few active members created risks, lower participation among other members was often due to personal and professional commitments rather than disillusionment with the network [62]. It is worth noting that WANEL’s current acquaintance network density of 19.5% falls within the range (15–25%) that threatens resilience and cohesion if strategically central actors exit the network [40, 41]. Furthermore, the overall structural pattern highlights WANEL’s limited network-level attributes, as observed in Mirzoev et al. The inadequacy of the network’s communication infrastructure and processes to facilitate broad-based engagement across linguistic boundaries is reflected in the network’s overreliance on a few bilingual actors. The critical bridging position held by actor BE01, which has configured the network into a hub and spoke [69], is a structural configuration that limits direct connections among members and restricts the development of trust-based relationships essential for knowledge sharing and collaboration [67, 70].

### Power dynamics and implications for inclusivity and equitable participation

The role of power dynamics in shaping access and participation in HPSR capacity-strengthening endeavours [20, 71, 72] and networks [73] has been documented. The EV4GH network, for instance, was widely praised for its commitment to amplifying under-represented voices within the HPSR space. However, the study identified several gaps and power differentials [62]: under-representation of certain language groups and geographies; a perceived “class bias”, with participants drawn disproportionately from privileged groups in LMICs; limited visibility and deliberate inclusion of Indigenous peoples and marginalized communities from high-income countries (HICs); low participation of individuals from LMIC institutions lacking strong HIC partnerships; and a bias towards candidates with pre-existing links to network partners. Similarly, while WANEL’s founding principles emphasized values such as transparency, mutual respect and professionalism, which mirror Mirzoev et al.’s normative framework, the network had no explicit strategy to address power dynamics. This may have allowed influence and power to concentrate informally among few actors, shaping access to resources and influencing the network’s trajectory. Krackhardt (1990) identified three forms of power in networks: structural (hierarchical positional), relational (centrality-based) and reputational (perceived influence) [74]. In WANEL, the disconnection between structural and relational power is reflected in the network’s distributed leadership where the formally appointed Technical Working Group (TWG) leader occupied a peripheral position, while another actor (BE01) held a central position, wielding considerable relational power (Fig. 2d). Additionally, the francophone–anglophone power imbalance which the founding leadership tried to avoid at the network’s inception has persisted. At the formation stage, the network aimed for a 50:50 representation but achieved a near balance of 55% and 45%, representation. Present membership however shows a reversal, with 72% anglophone and 28% francophone members. While HPSR is still an emerging field in Africa, it appears to be even less developed in francophone countries. The limited development of HPSR capacity in these contexts has been attributed to the scarcity of training opportunities and the lack of senior francophone HPSR actors to champion field-building efforts. These constraints are compounded by chronic underinvestment in HPSR [75] in general, but also the linguistic barriers that limit francophone HPSR actors’ access to funding opportunities [76]. Our findings show that the power dynamics reflected in centrality, influence and participation are more skewed towards anglophone actors today than they were at the beginning of WANEL. This indicates that the network’s failure to institutionalize its commitment to inclusivity and equitable participation created room for dominant groups to maintain control of relational and strategic influence. This again highlights Mirzoev et al.’s emphasis on the need for capacity-strengthening efforts to consciously surface the power dynamics operating within and between levels, prior to and during capacity-strengthening endeavours. Finally, the under-representation of francophone actors reflects the absence of a deliberate strategy to identify and engage capacity assets across the region. Notably, many francophone HPSR actors trained through the Emerging Voices for Global Health (EV4GH) program [7] were not integrated into WANEL. This missed opportunity highlights the importance of Mirzoev et al.’s framework’s recommendation that capacity-strengthening efforts must begin with comprehensive assessments that identify not only needs but also existing assets. Addressing these structural and relational gaps is essential for WANEL to evolve into a truly systemic capacity-strengthening platform.

### Study limitations

A few study limitations which are worth noting are discussed in continuation. Firstly, this SNA study did not achieve completeness of data for this socio-centric analysis. Potentially, the missing data resulting from the nine non-respondents could influence our findings, given the sensitivity of SNA to missing data. However, the study achieved a 94% response rate, which is substantially more than the 75% response rate that has been found to be the minimum threshold required for SNA data to be considered reliable [32]. Secondly, the study offers a cross-sectional examination of the interaction patterns in WANEL, but the participatory action research project within which this study is embedded allows for continuous cycles of reflection and implementation of actions. Thus, the recent implementation of an automated WANEL membership management system to facilitate connection may potentially lead to relational changes in the network, making it necessary for us to collect this data again after a year to examine the evolution. Finally, our analysis was based on unconfirmed ties, meaning the reciprocity of the relationships being reported was not considered a requirement. For this reason, we treated all the networks as directed networks and distinguished advice seekers (receivers) from the advice givers (sources). Even though confirmed data are considered a more reliable indicator of the existence of active relationship [77], the complimentary qualitative data we collected provided more explanation to the relational data in context (more details in our up-coming paper).

## Conclusion

WANEL represents a unique and promising initiative to strengthen HPSR capacity across West Africa. Its growth into a diverse network of over 100 members reflects increasing regional momentum to build a cross-country, cross-disciplinary learning community. With its mix of researchers, practitioners and policy actors, WANEL has significant amplification potential to translate individual capacity gains into broader system-level impacts. However, this potential is constrained by structural features such as low density, low cohesion and high centralization, which restrict equitable participation, hinder knowledge exchange and reinforce existing power imbalances. These dynamics not only threaten the effectiveness of WANEL as a knowledge translation and capacity-building platform but also place its sustainability at risk. To realize WANEL’s vision and strengthen its systemic role in HPSR capacity development, a strategic shift is needed, one that focuses not only on growing membership or increasing activities but on building relational infrastructure, distributing leadership and fostering inclusive engagement across all member groups. A strategic investment in building strong relational infrastructure, facilitated by regular, purposeful interaction across the diverse actors could make the network a resilient learning community that contributes meaningfully to HPSR field-building in West Africa. While several studies have documented empirical experiences of HPSR capacity assessments and strengthening efforts [14–19], a significant knowledge gap remains regarding the role of networking and network approach to capacity-building, despite the existence of several regional HPSR networks [78]. This study is among the very few [79] that contribute further insight into understanding how networks function as mechanisms for HPSR capacity-strengthening.

## Supplementary Information

Electronic Supplementary Material 1.

Electronic Supplementary Material 2.

Electronic Supplementary Material 3.

## Data Availability

The datasets generated and/or analysed during the current study are not publicly available due to the possibility of identifying individuals who participated but are available from the corresponding author on reasonable request.

## Abbreviations

EV4GH: Emerging Voices for Global Health
SNA: Social Network Analysis
WANEL: West African Network of Emerging Leaders in Health Policy and Systems Research
HPSR: Health policy and systems research
LMICs: Low- and middle-income countries
TWGs: Thematic Working Groups
M&E: Monitoring and Evaluation

## Footnotes

1 Defor et al., “Networking as a pathway to health policy and systems research capacity building in LMICs: The example of the West African Network of Emerging Leaders in HPSR.” (Unpublished).

2 Defor et al., “Networking as a pathway to health policy and systems research capacity building in LMICs: The example of the West African Network of Emerging Leaders in HPSR.” (Unpublished).
