# Supplementary material for: Peer-learning and support among health policy and systems research actors in West Africa: a social network analysis
Source: Health Res Policy Syst. 2025 Nov 13;23:151. doi: 10.1186/s12961-025-01417-6 (PMC12613594; doi:10.1186/s12961-025-01417-6)

**Article title: Peer-learning and support among Health Policy and Systems Research actors in West Africa: A social network analysis**

**Author’s information:** Selina Defor^1, 2,^ Fidele Kanyimbu Mukinda^1^, Fadima Yaya Bocoum^2^, Ermel Johnson**^2,^** Irene A. Agyepong ^3^ and Uta Lehmann^1^

1School of Public Health, University of the Western Cape, Cape Town, South Africa

2West African Network of Emerging Leaders in Health Policy and Systems (WANEL)

3Public Health Faculty, Ghana College of Physicians and Surgeons

**Corresponding author:** Selina Defor E-mail: sellydel@yahoo.com

**Additional file 1 WANEL Country connectivity**

**Figure 1 Cross-country and intra-country acquaintance connection before and after WANEL membership**


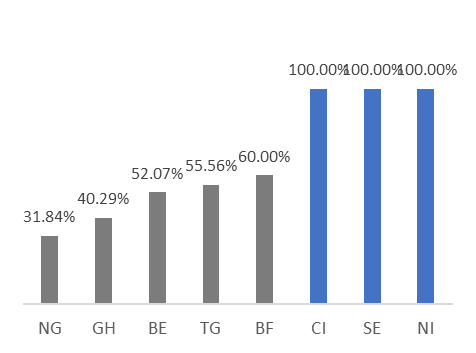


Figure 1b: Intra-Country Connectivity (after WANEL )

(Prior WANEL)


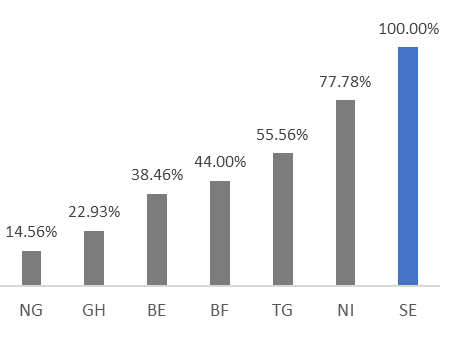


Figure 1a: Intra-Country Connections (before WANEL)


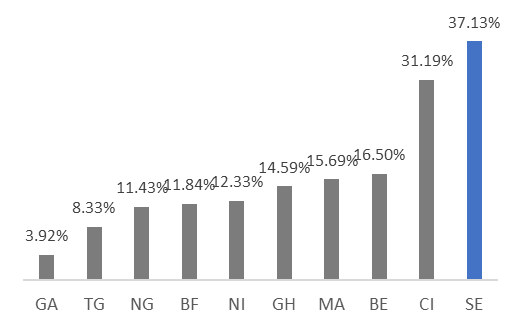


Figure 1d: Cross-Country Connections (after WANEL)

Figure 1c: Cross-Country Connections (before WANEL)


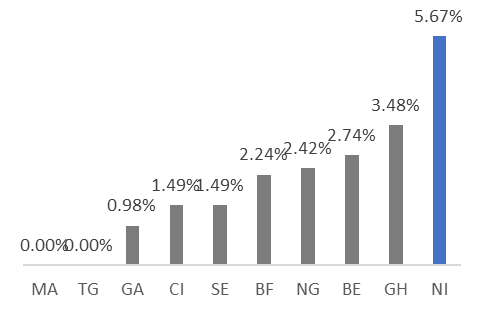


**Figure 2 Betweenness centrality scores of the first 30 WANEL members**


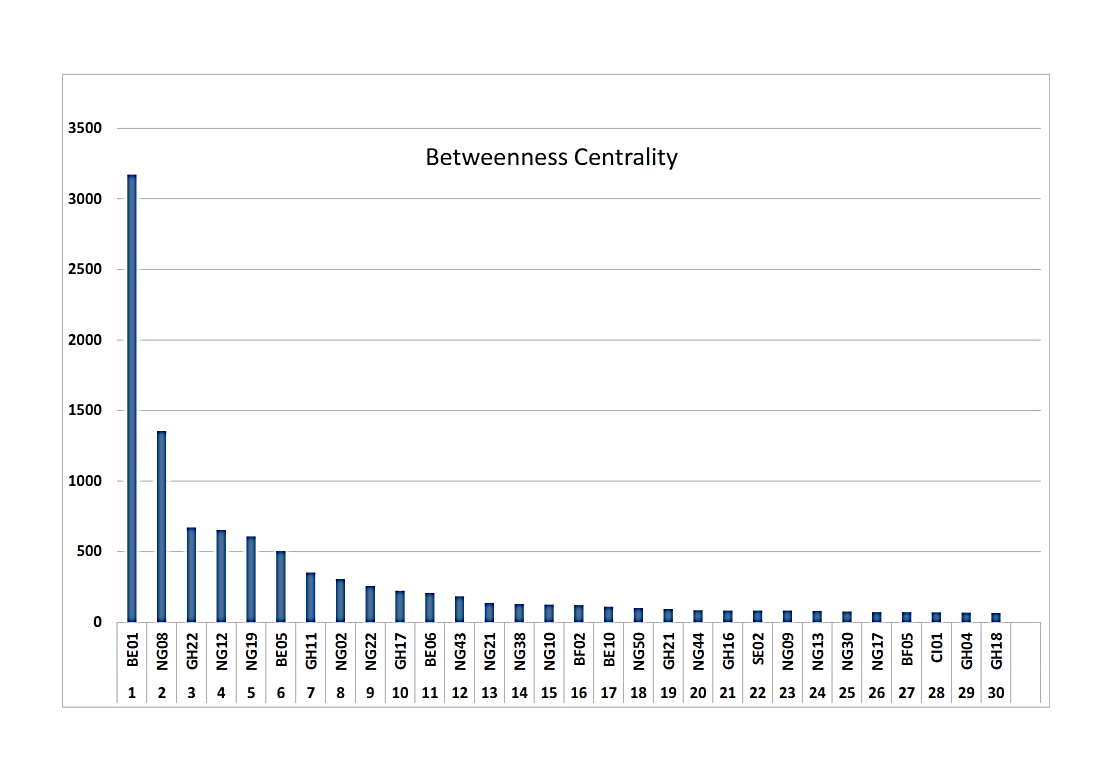

Supplement: Supplementary file 1 — Electronic Supplementary Material 1. [file 12961_2025_1417_MOESM1_ESM.docx]
