# Supplementary material for: Peer-learning and support among health policy and systems research actors in West Africa: a social network analysis
Source: Health Res Policy Syst. 2025 Nov 13;23:151. doi: 10.1186/s12961-025-01417-6 (PMC12613594; doi:10.1186/s12961-025-01417-6)
Supplement: Supplementary file 2 — Electronic Supplementary Material 2. [file 12961_2025_1417_MOESM2_ESM.docx]

**Article title: Peer-learning and support among Health Policy and Systems Research actors in West Africa: A social network analysis**

**Author’s information:** Selina Defor^1, 2,^ Fidele Kanyimbu Mukinda^1^, Fadima Yaya Bocoum^2^, Ermel Johnson**^2,^** Irene A. Agyepong ^3^ and Uta Lehmann^1^

1School of Public Health, University of the Western Cape, Cape Town, South Africa

2West African Network of Emerging Leaders in Health Policy and Systems (WANEL)

3Public Health Faculty, Ghana College of Physicians and Surgeons

**Corresponding author:** Selina Defor E-mail: sellydel@yahoo.com

**Additional file 2: Cross-disciplinary collaboration and advice sharing**

**Additional file 2 :** **Cross-disciplinary collaboration and advice sharing**

Fig 1a cross -disciplinary collaboration network


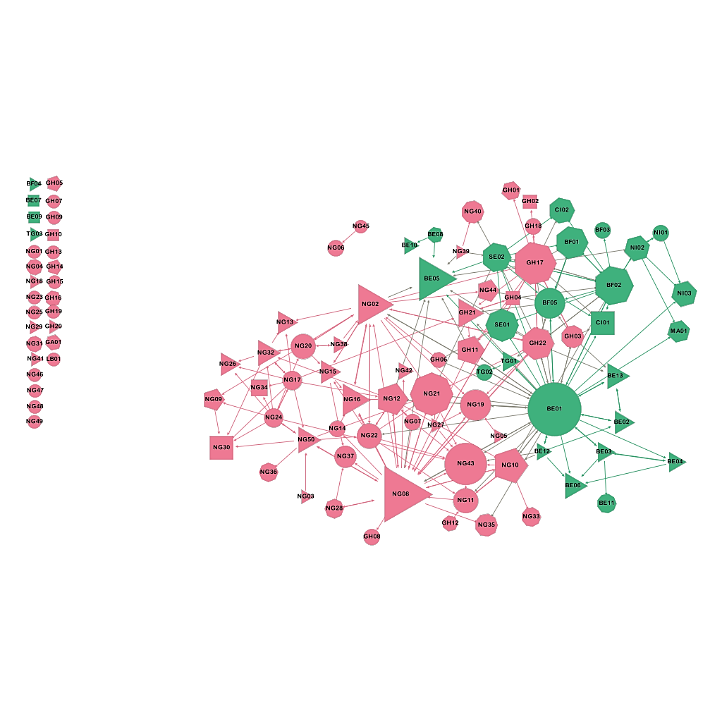


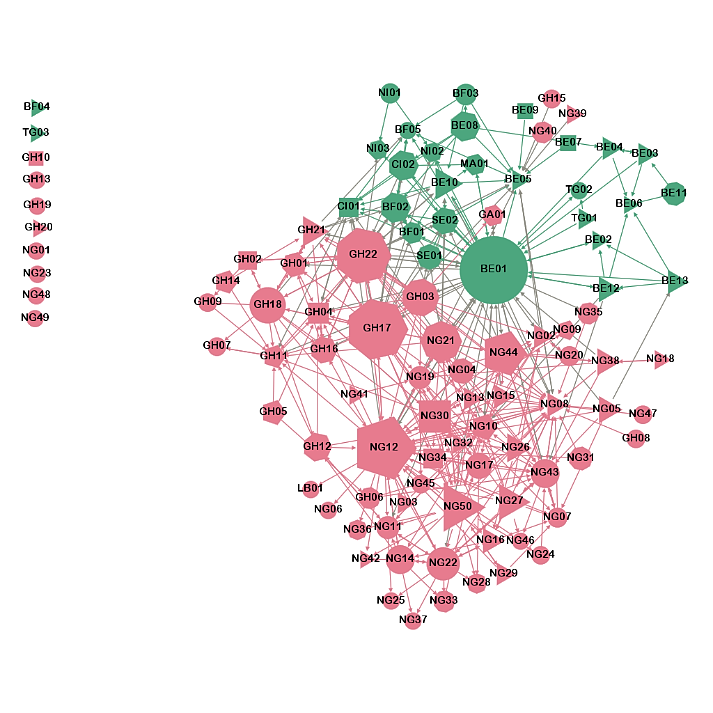
Fig 1b cross -disciplinary advice network
