# Supplementary material for: Peer-learning and support among health policy and systems research actors in West Africa: a social network analysis
Source: Health Res Policy Syst. 2025 Nov 13;23:151. doi: 10.1186/s12961-025-01417-6 (PMC12613594; doi:10.1186/s12961-025-01417-6)
Supplement: Supplementary file 3 — Electronic Supplementary Material 3. [file 12961_2025_1417_MOESM3_ESM.docx]

**Article title: Peer-learning and support among Health Policy and Systems Research actors in West Africa: A social network analysis**

**Author’s information:** Selina Defor^1, 2,^ Fidele Kanyimbu Mukinda^1^, Fadima Yaya Bocoum^2^, Ermel Johnson**^2,^** Irene A. Agyepong ^3^ and Uta Lehmann^1^

1School of Public Health, University of the Western Cape, Cape Town, South Africa

2West African Network of Emerging Leaders in Health Policy and Systems (WANEL)

3Public Health Faculty, Ghana College of Physicians and Surgeons

**Corresponding author:** Selina Defor E-mail: sellydel@yahoo.com

**Additional file 3:** Network isolates

**Table 1** Characteristics of isolates in the Mentorship, Professional advice, and collaboration networks

| **Mentorship** | |  |  | **Professional Advice** | |  |  | **Disciplinary Collaboration** | | | |
| --- | --- | --- | --- | --- | --- | --- | --- | --- | --- | --- | --- |
|  |  |  |  |  |  |  |  |  |  | |  |
| Age |  |  |  | Age |  |  |  | Age |  | |  |
| 31 to 40 | 13 | 56.52% |  | 31 to 40 | 5 | 71.43% |  | 31 to 40 | 16 | | 69.57% |
| 41 to 50 | 6 | 26.09% |  | 41 to 50 | 1 | 14.29% |  | 41 to 50 | 3 | | 13.04% |
| 20 to 30 | 3 | 13.04% |  | 51 to 60 | 1 | 14.29% |  | 20 to 30 | 2 | | 8.70% |
| 51 to 60 | 1 | 4.35% |  |  |  |  |  | 51 to 60 | 2 | | 8.70% |
|  |  |  |  |  |  |  |  |  |  | |  |
| **Educational background** | | |  | **Educational background** | | |  | **Educational background** | | | |
| Masters | 18 | 62.07% |  | PhD | 5 | 50.00% |  | Masters | 16 | | 57.14% |
| PhD | 9 | 31.03% |  | Masters | 4 | 40.00% |  | PhD | 10 | | 35.71% |
| Bachelors | 2 | 6.90% |  | Bachelors | 1 | 10.00% |  | Bachelors | 2 | | 7.14% |
|  |  |  |  |  |  |  |  |  |  | |  |
| **Profession** | |  |  | **Profession** | |  |  | **Profession** | |  | |
| Non– medical practitioner | 8 | 27.59% |  | Post-doctoral researcher | 3 | 30.00% |  | Medical practitioner | 6 | | 21.43% |
| Researcher | 7 | 24.14% |  | Medical practitioner | 2 | 20.00% |  | Doctoral researcher | 5 | | 17.86% |
| Medical practitioner | 5 | 17.24% |  | Researcher | 2 | 20.00% |  | Non– medical practitioner | 5 | | 17.86% |
| Doctoral researcher | 3 | 10.34% |  | Doctoral researcher | 1 | 10.00% |  | Researcher | 5 | | 17.86% |
| Post– doctoral researcher | 3 | 10.34% |  | Non-medical practitioner | 1 | 10.00% |  | Post– doctoral researcher | 4 | | 14.29% |
| Lecturer | 2 | 6.90% |  | Unemployed | 1 | 10.00% |  | Lecturer | 2 | | 7.14% |
| Unemployed | 1 | 3.45% |  |  |  |  |  | Unemployed | 1 | | 3.57% |
|  |  |  |  |  |  |  |  |  |  | |  |
| **Years of professional experience** | | | | **Years of professional experience** | | | | **Years of professional experience** | | | |
| 4 – 7 years | 12 | 42.86% |  | 1 – 3 years | 4 | 44.44% |  | 4 – 7 years | 13 | | 48.15% |
| 1 – 3 years | 7 | 25.00% |  | 4 – 7 years | 4 | 44.44% |  | 1 – 3 years | 10 | | 37.04% |
| 8 – 10 years | 4 | 14.29% |  | Over 10 years | 1 | 11.11% |  | 8 – 10 years | 2 | | 7.41% |
| Over 10 years | 3 | 10.71% |  |  |  |  |  | Over 10 years | 2 | | 7.41% |
| 6 months to less than 1 year | 1 | 3.57% |  |  |  |  |  |  |  | |  |
| Less than 6 months | 1 | 3.57% |  |  |  |  |  |  |  | |  |
|  |  |  |  |  |  |  |  |  |  | |  |
| **Membership in related HPSR network** | | | | **Membership in related HPSR network** | | | | **Membership in related HPSR network** | | | |
| Yes | 9 | 40.91% |  | Yes | 3 | 50.00% |  | Yes | 9 | | 40.91% |
| No | 13 | 59.09% |  | No | 3 | 50.00% |  | No | 13 | | 59.09% |
